# Effectiveness of an Advanced Clinical Decision Support System on Clinical Decision-Making Skills in a Call Center Medication Therapy Management Pharmacy Setting: A Pilot Study

**DOI:** 10.3390/pharmacy8040228

**Published:** 2020-11-25

**Authors:** Jennifer M. Bingham, Veronique Michaud, Jacques Turgeon, David R. Axon

**Affiliations:** 1 Applied Precision Pharmacotherapy Institute, Tabula Rasa HealthCare, Tucson, AZ 85701, USA; jbingham@trhc.com; 2 Precision Pharmacotherapy Research & Development Institute, Tabula Rasa HealthCare, Lake Nona, FL 32827, USA; vmichaud@trhc.com (V.M.); jturgeon@trhc.com (J.T.); 3 Department of Pharmaceutical Sciences, College of Pharmacy, University of Arizona, Tucson, AZ 85721, USA

**Keywords:** medication therapy management, clinical decision support systems, training, pharmacist

## Abstract

(1) Background: There is limited evidence related to the efficacy of advanced clinical decision support systems (CDSS) on the quantity of high-quality clinical recommendations in a pharmacy-related medication therapy management (MTM) setting. The study aimed to assess the effect of an advanced CDSS on the quantity of relevant clinical pharmacist recommendations in a call center MTM setting. (2) Methods: This pre-test/post-test with comparator group study compared clinical skills assessment scores between certified MTM pharmacists in March 2020. A Wilcoxon Signed Rank test assessed the difference between pre- and post-test scores in both groups. (3) Results: Of 20 participants, the majority were less than 40 years old (85%) with a Doctor of Pharmacy degree (90%). Nine were female. Intervention group participants had less than three years of experience as a pharmacist. The control group had less than three years (40%) or seven to ten years (40%) of experience. There was a significant increase in intervention group scores between pre- (median = 3.0, IQR = 3.0) and post-test segments (median = 6.5, IQR = 4.0, *p* = 0.02). There was no significant change between control group pre- and post-test segments (*p* = 0.48). (4) Conclusion: Pharmacist exposure to an advanced CDSS was associated with significantly increased quantity of relevant clinical recommendations in an MTM pharmacy setting.

## 1. Introduction

Polypharmacy is associated with a higher risk of drug-drug interactions and adverse drug events (ADEs) [1]. Pharmacists play a key role in the prevention and detection of drug-drug interactions (DDI) [2]. It is critical for pharmacists to intervene and improve the medication safety profile of drug regimens for patients with multiple chronic diseases and polypharmacy; yet, few clinical tools are available to accurately assess these complex regimens for interactions and ADEs [3].

Clinical decision support systems (CDSS) improve patient safety [4], and alert providers about potentially dangerous DDIs. These systems provide a plethora of evidence shown to improve the process of care and reduce medication errors [5]. Nonetheless, interpretation by healthcare providers can be difficult when there is polypharmacy [6]. Despite an abundance of information, healthcare providers cannot always rely on the clinical relevance of these commercially available CDSS because they do not consider medication timing, medication dose, and patient comorbidities [6].

Most drug interaction screening software systems (DISS) only compare one medication to another, whereas advanced CDSS provides more extensive and meaningful opportunities to improve medication safety by simultaneously analyzing DDI information on all medications in the medication regimen [6,7]. MedWise^™^ is an advanced CDSS platform for pharmacists, which takes into consideration numerous medication characteristics, including cytochrome P450 drug/gene, drug/drug/gene, and drug/disease/gene interactions by specific isoforms, in addition to risk of drug-induced long QT syndrome, anticholinergic burden, sedative burden (Patent No. US10,720,241), and overall relative odds ratio for adverse drug events [8]. This inclusive CDSS also incorporates patient characteristics, including age, gender, renal function, laboratory results, allergies, and pharmacogenomic results [8]. In 2019, a novel systematic training program was created specific to the CDSS (MedWise^™^, Moorestown, NJ, USA). Yet, little is known about whether access to an advanced CDSS combined with a training program increases the quantity of relevant clinical recommendations provided by certified medication therapy management (MTM) pharmacists.

To address this gap in the literature, a pre-test/post-test study was developed to assess the effectiveness of the CDSS and its training program in an MTM pharmacy setting. The study objective was to determine whether exposure to the advanced CDSS, in conjunction with the novel systematic training program, improved the quantity of relevant medication safety-related recommendations made by the pharmacist.

## 2. Materials and Methods

### 2.1. Study Design

This pilot study employed a pre-test/post-test with comparator group design to compare the effect of a clinical skills case assessment among 20 certified MTM pharmacists at two locations. One medication therapy management pharmacist call center location served as the intervention site (Florida) and the other served as the control site (Arizona). This project was approved by the University of Arizona institutional review board (No. 2001337677).

### 2.2. Study Participants and Site

The national MTM call center provides a suite of MTM services to meet the performance needs of health plans and patients, mainly through pharmacist-provided telehealth medication reviews. The center consists of a team of pharmacists dedicated to improving health, wellness, and chronic disease management through MTM services adopting an interprofessional team model that included pharmacy technicians, student pharmacists, pharmacy residents, nursing students, and registered nurses. MTM pharmacists at the call center provide telephonic comprehensive and targeted medication reviews to eligible Medicare beneficiaries. The pharmacists reconcile medication lists and review adherence to national consensus treatment guidelines, medication nonadherence, dosing concerns, drug-drug interactions, and high-risk medication use. The pharmacists use their clinical expertise and access to DISS to complete this task.

### 2.3. Study Recruitment and Enrollment

Participants were included in the study if they were 18 years of age or older and employed as a pharmacist at the MTM call center. The principal investigator distributed a recruitment email in February 2020 to two MTM center pharmacy directors, which was forwarded to 29 eligible pharmacists at their sites. The email provided information about the study and invited pharmacists to participate by responding directly to the principal investigator.

**Figure 1:**
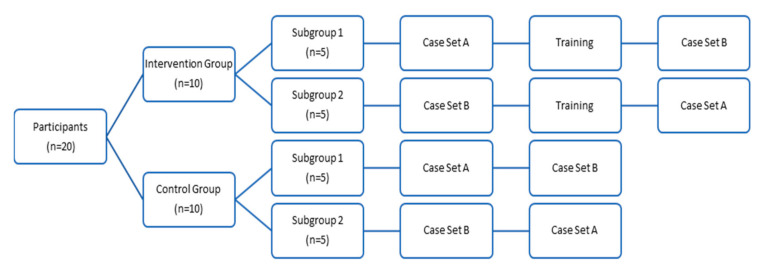
Overview of pre-test/post-test segments with comparator group study design.

The principal investigator randomly assigned the first ten enrolled participants at each site into one of two subgroups using a blocked randomization approach, which determined the order in which participants would complete their two sets of clinical skill assessment segments. There were two case sets (A and B) that each contained three clinical cases, which were similar in terms of complexity. Both subgroups were blinded prior to the clinical case assessment segments. Subgroup 1 completed case set A first, and case set B second; while subgroup 2 completed case set B first, and case set A second (Figure 1). See Appendix A for a sample of cases used in Clinical Case Set A and Appendix B for a sample of cases used in Clinical Case Assessment B.

### 2.4. Clinical Skills Assessment

The clinical skills assessment was conducted using Survey Monkey [9] and constituted the pre-test/post-test in this study. Participants were required to make clinical recommendations on each case using the following Systematized Nomenclature of Medicine—Clinical Terms (SNOMED-CT): recommendation to change medication; recommendation to decrease medication dose; recommendation to increase medication dose; recommendation to change medication timing of administration; recommendation to start medication; recommendation to discontinue medication; recommendation to start monitoring [10]. Participants selected SNOMED-CTs that corresponded to their recommendations, and elaborated on their recommendation with open text.

### 2.5. Intervention

The study intervention was provided to the intervention group over two days. The first part consisted of eight hours of online training on the proprietary CDSS. The training program consisted of six modules and encompassed multiple concepts, including: competitive inhibition drug-drug interaction, anticholinergic risk, sedative risk, long QT syndrome, pharmacokinetic case examples, and medication risk mitigation. The second part consisted of two hours of live training with two CDSS subject matter experts. The live session consisted of interactive case discussions to further develop understanding of basic concepts related to the CDSS.

### 2.6. Data Collection

#### 2.6.1. Intervention Group

Data collection for the intervention group took place over two full business days (7 and 8 March 2020). On day one, participants in the intervention group were sent an email containing instructions and a link to complete the pre-test clinical skill assessment segment. Subgroup 1 completed case set A, while subgroup 2 completed case set B. Then, all participants received the online training intervention. On day two, the intervention continued with all participants receiving the live training session. Then, participants received an email with the next set of instructions and a link to complete the post-test clinical skill assessment segment. Subgroup 1 completed case set B, while subgroup 2 completed case set A. Participants in the intervention group had access to all usual paired DDI tools typically used in clinical practice, both in the pre- and post-test situations. In addition, they had access to the advanced CDSS (MedWise^™^) in the post-test situation. Finally, both subgroups completed an online questionnaire asking about their demographic characteristics (age, gender, pharmacy practice setting, years of experience as a pharmacist, and credentials) and their perception of the CDSS (Did you find the clinical decision support system to be helpful?, Would you recommend the decision support system to others?, Would use of a clinical decision support system improve your quantity of clinical recommendations?, Would use of a clinical decision support system improve your quality of clinical recommendations?, Would use of a clinical decision support system enable you to provide better patient care?). Response options for the perception assessments included strongly agree, agree, disagree, and strongly disagree.

#### 2.6.2. Control Group

Data collection for the control group took place in one business day (7 March 2020). Similar to the intervention group, participants in the control group were sent an email containing instructions and a link to complete the pre-test clinical skills assessment segment. Subgroup 1 completed case set A, while subgroup 2 completed case set B. As there was no training on the CDSS for the control group, participants then received an email with instructions and a link to complete the post-test clinical skills assessment. Subgroup 1 completed case set B, while subgroup 2 completed case set A. Participants in the control group also had access to the usual paired DDI tools used in clinical practice when they completed both case sets. Participants also completed the same questionnaire about demographics and perceptions of an advanced CDSS as the intervention group, with the exception of two items (Did you find the clinical decision support system to be helpful?, Would you recommend the decision support system to others?).

### 2.7. Data Analysis

Case sets were scored based on correct responses according to a standardized answer key provided by two blinded CDSS subject matter experts to avoid bias. These reviewers were not aware of the participant groups and to which order case sets A and B were answered. Participants received one point for every correct response. Descriptive statistics were used to describe the study participants. Wilcoxon Signed Rank tests (non-parametric tests were used due to the skewed distribution of data) were computed to compare the pre- and post-test values in both the intervention and control groups. All analyses were conducted using SAS University Edition^™^ (Cary, NC, USA). An a priori alpha level of 0.05 was used.

## 3. Results

The study sample consisted of 20 participants who were certified MTM pharmacists. The majority were less than 40 years of age (85%), and gender was approximately evenly split between males and females. Participants worked in a variety of settings: community pharmacy (*n* = 10), ambulatory care pharmacy (*n* = 7), and managed care pharmacy (*n* = 7). In the intervention group, participants typically had less than three years of experience as a pharmacist, while control group participants’ experience as a pharmacist most commonly was less than three years (40%) or seven to ten years (40%). In both groups, 90% of participants held a Doctor of Pharmacy (PharmD) degree, while 10% held a Bachelor of Pharmacy (BPharm) degree. One intervention group participant also held a Master of Public Health (MPH) degree (Table 1).

All participants in the intervention group strongly agreed that the CDSS was helpful and would recommend it to others. All participants strongly agreed or agreed that the use of a CDSS would help improve the quantity and quality of their clinical recommendations and enable them to provide better patient care (Table 2).

In the intervention group, there was a significant increase in scores between pre-test and post-test segments from a median of 3.0 (IQR = 3.0) to a median of 6.5 (IQR = 4.0, *p* = 0.02). However, in the control group, there was no significant change between pre-test (median = 3.0, IQR = 2.0) and post-test segments (median = 3.5, IQR = 1.0, *p* = 0.48). See Table 3 for further details.

## 4. Discussion

The study results support a role for advanced CDSS accompanied with a systematic training program in an MTM pharmacy setting. Pharmacists who used the CDSS and completed the training had improved clinical assessment scores. The results showed that pharmacists exposed to an advanced CDSS and associated training had improved quality of clinical recommendations, whereas those who used traditional paired DDI tools did not. The association between an advanced CDSS and quality of pharmacist medication safety-related recommendations was not previously evaluated. While a few studies report improved clinical outcomes associated with CDSS [5,8], none discuss the effect of an advanced CDSS (MedWise^™^) combined with a systematic training program on an MTM pharmacist’s recommendations and on overall clinical decision-making skills.

Most participants in both intervention and control pre-test groups selected the SNOMED-CT, recommendation to start a medication. The investigators thus inferred that pharmacists who were not exposed to an advanced CDSS were more inclined to adhere to national consensus guideline recommendations based on previous ambulatory care pharmacy experience at the call center. Instead, the intervention post-group selected: recommendation to change medication; recommendation to change medication timing of administration; recommendation to discontinue medication; and recommendation to start monitoring. The results suggest that pharmacists who used the CDSS and participated in the training were better equipped to provide personalized clinical recommendations as a direct result of their exposure to principles outlining the importance of and correct processes for clinically assessing medication dose, medication, and comorbidities [6].

The intervention post-test group also recommended more sequential actionable items that coincided with the SNOMED-CT. These recommendations were made to prevent further medication safety-related concerns in the simulated case assessments compared to the control group. This suggests a need to integrate advanced CDSS in MTM pharmacy settings to help pharmacists to minimize risk of potential ADEs. This is consistent with the results of a study that demonstrated how evidence-based guidance and medication risk scores help pharmacists recommend more appropriate use of medications, avoiding ADEs and medication-related morbidity [11]. The results also highlight the benefits of CDSS to improve the process of care, including pharmacist performance, as demonstrated in another study [5].

One strength of this study is that it shows that an advanced CDSS, layered with a systematic training program, can aid pharmacist in avoiding inappropriate use of drugs, ADEs, and polypharmacy. It demonstrates that pharmacists exposed to the advanced CDSS are more equipped to provide accurate, evidence-based clinical recommendations, compared to pharmacists who solely relied on DISS in the call center MTM setting. It supports the need for pharmacists to comprehend the effects of DDIs affected by cytochrome P450 (CYP) on medication pharmacokinetics and patient response [12]. It further supports the value in assessing CYP DDIs to predict clinical outcomes [13]. Perhaps most significantly, it also highlights previous research conclusions that advanced CDSS can help pharmacists to quickly and easily synthesize pharmacokinetics/pharmacodynamics drug properties, multi-drug interactions, pharmacogenetics, efficacy, and toxicity of drug ingredients to improve medication safety and reduce ADEs [6,11].

### Limitations

Despite the significantly improved quality of clinical assessment responses, this study only utilized one national MTM provider group of pharmacists and was limited by a small sample size. The study did not control for the type of DISS that was used in both the control and intervention groups. The study also did not capture outcomes for board of pharmacy specialty certified pharmacists, as there were no Board of Pharmacy Specialties-certified pharmacists enrolled in the study after randomization. Thus, these findings are not generalizable to all pharmacists practicing in a call center MTM setting.

## 5. Conclusions

Advanced CDSS access, preceded by a systematic training program, was successful in increasing the quantity of relevant clinician recommendations in a clinical case assessment. Future consideration towards the implementation of advanced CDSS with appropriate systematic training programs in other pharmacy settings is warranted.

## Figures and Tables

**Table 1:** Characteristics of study participants in the intervention and control groups.

Characteristic	Intervention Group (*n* = 10) *n* (%)	Control Group (*n* = 10) *n* (%)
Age (years)		
20–30	8 (80)	2 (20)
31–40	1 (10)	6 (60)
41–50	0	1 (10)
51–60	1 (10)	1 (10)
Female gender	5 (50)	4 (40)
Pharmacy Practice Setting		
Community Pharmacy	6 (60)	4 (40)
Hospital Pharmacy	1 (10)	0
Ambulatory Care Pharmacy	1 (10)	6 (60)
Managed Care Pharmacy	6 (60)	1 (10)
Other	3 (30)	0
Years of Experience as a Pharmacist		
0–3	7 (70)	4 (40)
4–6	1 (10)	1 (10)
7–10	0	4 (40)
10 or more	2 (20)	1 (10)
Credentials		
BPharm	1 (10)	1 (10)
PharmD	9 (90)	9 (90)
MPH	1 (10)	0

Key: BPharm = Bachelor of Pharmacy; PharmD = Doctor of Pharmacy; MPH = Master of Public Health.

**Table 2:** Subject Perception of the Advanced Clinical Decision Support System Post Systematic Training (CDSS).

Characteristic	Intervention Group (*n* = 10) *n* (%)	Control Group (*n* = 10) *n* (%)
“Did you find the clinical decision support system to be helpful?”		
Strongly Agree	10 (100)	N/A
“Would you recommend the decision support system to others?”		
Strongly Agree	10 (100)	N/A
“Would use of a clinical decision support system improve your quantity of clinical recommendations?”		
Strongly Agree	8 (80)	6 (60)
Agree	2 (20)	4 (40)
“Would use of a clinical decision support system improve your quality of clinical recommendations?”		
Strongly Agree	10 (100)	6 (60)
Agree	0	4 (40)
*“*Would use of a clinical decision support system enable you to provide better patient care?”		
Strongly Agree	10 (100)	5 (50)
Agree	0	5 (50)

Key: N/A = not applicable.

**Table 3:** Change in pre- and post-test median scores in the intervention and control groups.

	Pre-Test, Median (IQR)	Post-Test, Median (IQR)	*p*-Value
**Intervention group**	3.0 (3.0)	6.5 (4.0)	0.02
**Control group**	3.0 (2.0)	3.5 (1.0)	0.48

Key: IQR = interquartile range

## Appendix A: Clinical Assessment Case Set A

1. Your patient is a 76 y/o Male with NKDA. SCr 0.98, Height 6′1”, Weight 251 lb. Past medical history includes: coronary artery disease, anxiety disorder, gout, hyperlipidemia, insomnia, low testosterone, sleep apnea, vitamin D deficiency.
  He is taking:
    Allopurinol 300 mg 2 tabs PO qAM
    Aspirin 325 mg PO qAM
    Bupropion XL 150 mg PO qAM
    Clopidogrel 75 mg PO qAM
    Escitalopram 20 mg PO qAM
    Metoprolol Tartrate 25 mg PO BID
    Omega 3 (strength unknown) 1 PO BID
    Oxycodone-Acetaminophen 5–325 mg PO q4h prn
    Rosuvastatin 40 mg PO qAM
    Zolpidem 10 mg PO qHS

**Select your clinical recommendation(s) using the following options (SELECT ALL THAT APPLY):**

□ Recommendation to change medication
□ Recommendation to decrease medication dose
□ Recommendation to increase medication dose
□ Recommendation to change medication timing of administration
□ Recommendation to start medication
□ Recommendation to discontinue medication
□ Recommendation to start monitoring

In the space below, SPECIFY the medication name that coincides with your recommendations noted above:

2. Your patient (age and gender unknown) has the following labs: Mg 2.0, K 4.4. Allergic to penicillin. Past medical history includes: atrial flutter with rapid ventricular response, coronary artery disease status post stent, deep vein thrombosis, hypertension, hypothyroidism.
  They are taking:
    Lisinopril 10 mg PO qAM
    Loratadine 10 mg PO qAM
    Levothyroxine 88 mcg PO qAM
    Metoprolol ER 50 mg PO qAM
    Warfarin 2.5 mg PO qAM
    Sertraline 25 mg PO qAM
    Venlafaxine ER 150 mg PO qAM
    Vitamin D 1000 international units PO qAM
    Pantoprazole 40 mg PO qAM
    Atorvastatin 40 mg PO qAM
    Clopidogrel 75 mg PO qAM
    Ferrous sulfate 325 mg PO qAM
    Potassium chloride 20 meq PO qAM
    Amiodarone 400 mg PO BID
    Digoxin 125 mcg PO qAM

**Select your clinical recommendation(s) using the following options (SELECT ALL THAT APPLY):**

□ Recommendation to change medication
□ Recommendation to decrease medication dose
□ Recommendation to increase medication dose
□ Recommendation to change medication timing of administration
□ Recommendation to start medication
□ Recommendation to discontinue medication
□ Recommendation to start monitoring

In the space below, SPECIFY the medication name that coincides with your recommendations noted above:

3. Your patient is a 73 y/o female that is allergic to alprazolam. Labs = Mg 2.0, K 4.4. Past medical history includes: Trigeminal neuralgia, major depressive disorder, chronic obstructive pulmonary disease (stage 3) with multiple hospital admissions, history of SIADH.
  She is taking:
    Aspirin 81 mg PO qAM
    Calcichew PO qAM
    Lisinopril 20 mg PO qAM
    Carbamazepine 200 mg PO qAM
    Terazosin 4 mg PO qAM
    Zolpidem 10 mg PO qHS prn
    Mirtazapine 30 mg PO qHS + 7.5 mg PO qHS prn
    Famotidine 20 mg PO BID
    Senna 8.6 mg PO BID
    Amlodipine 10 mg PO qAM
    Pregabalin 75 mg PO BID
    Albuterol Inhale 2 puffs PO 5 times daily +4 puffs q4h prn
    Tiotropium Respimat 2 puffs PO daily

**Select your clinical recommendation(s) using the following options (SELECT ALL THAT APPLY):**

□ Recommendation to change medication
□ Recommendation to decrease medication dose
□ Recommendation to increase medication dose
□ Recommendation to change medication timing of administration
□ Recommendation to start medication
□ Recommendation to discontinue medication
□ Recommendation to start monitoring

In the space below, SPECIFY the medication name that coincides with your recommendations noted above:

## Appendix B: Clinical Assessment Case Set B

1. Your patient is an 82-year-old male that is allergic to dye. His Scr is 1.1, Height 5’2”, 190 lb. Past medical history includes: STEMI 2016, chronic stable angina, chronic low back pain, BPH, GERD, hypothyroidism.
  He is taking:
    Acetaminophen 500 mg PO TID
    Aspirin 81 mg PO qAM
    Atorvastatin 40 mg PO qHS
    Calcium carbonate 600 mg PO BID
    Cholecalciferol 1000 unit PO qAM
    Clopidogrel 75 mg tablet PO qAM
    Docusate sodium 100 mg PO qAM
    Esomeprazole 40 mg PO qAM
    Famotidine 10 mg PO qAM
    Ferrous sulfate 325 mg PO BID
    Finasteride 5 mg PO qAM
    Isosorbide mononitrate ER 30 mg PO qAM
    Levothyroxine 112 mcg PO qAM
    Metoprolol tartrate 25 mg PO BID
    Oxycodone-acetaminophen 7.5 mg/325 mg PO TID prn
    Polyethylene glycol 3350 17 g/dose PO qAM
    Tamsulosin 0.4 mg PO qAM

**Select your clinical recommendation(s) using the following options (SELECT ALL THAT APPLY):**

□ Recommendation to change medication
□ Recommendation to decrease medication dose
□ Recommendation to increase medication dose
□ Recommendation to change medication timing of administration
□ Recommendation to start medication
□ Recommendation to discontinue medication
□ Recommendation to start monitoring

In the space below, SPECIFY the medication name/parameter that coincides with your recommendations noted above:

2. Your patient is a 63 y/o female with NKDA. No recent labs. Her past medical history includes: hypertension, hyperlipidemia, diabetes mellitus, peripheral neuropathy, essential tremor, depression, anxiety, history cardiovascular accident, carotid stenosis, irritable bowel syndrome, history of falls.
  She is taking:
    Acetaminophen 325 mg tablet 2 PO q6h prn pain
    Aspirin 81 mg, delayed release PO qAM
    Clopidogrel 75 mg PO qAM
    Fluoxetine 20 mg PO qAM
    Folic acid 1 mg PO qAM
    Gabapentin 300 mg PO qAM
    Lisinopril 5 mg PO qAM
    Loperamide 2 mg PO daily prn, up to 2 tabs per day
    Melatonin 3 mg tablet PO qHS
    Metformin 500 mg PO BID with meals
    Mirtazapine 7.5 mg PO qHS prn
    Pantoprazole 40 mg, delayed release PO qAM
    Propranolol 60 mg tablet 1.5 PO BID
    Thiamine HCl 100 mg PO qAM

**Select your clinical recommendation(s) using the following options (SELECT ALL THAT APPLY):**

□ Recommendation to change medication
□ Recommendation to decrease medication dose
□ Recommendation to increase medication dose
□ Recommendation to change medication timing of administration
□ Recommendation to start medication
□ Recommendation to discontinue medication
□ Recommendation to start monitoring

In the space below, SPECIFY the medication name/parameter that coincides with your recommendations noted above:

3. Your patient is an 81 y/o Female, NKDA with a past medical history of anxiety, atrial fibrillation, gastroesophageal reflux disease, essential tremors, hypertension, hypothyroidism.
  She is taking:
    Acetaminophen 325 mg PO TID
    Lactobacillus 10 billion cell PO qAM
    Diltiazem ER 360 mg 24 h PO qAM
    Dulera 100 mcg-5 mcg/actuation HFA aerosol inhaler 2 puffs PO BID
    Duloxetine 60 mg, delayed release PO qAM
    Levothyroxine 88 mcg capsule PO qAM
    Lorazepam 0.5 mg PO TID
    Ipratropium-albuterol 0.5 mg–3 mg (2.5 mg base)/3 mL inhaled q6h prn
    Pantoprazole 40 mg, delayed release PO qAM
    Potassium chloride ER 20 mEq, extended release PO qAM
    Quetiapine 25 mg PO qAM
    Topiramate 50 mg PO qAm

**Select your clinical recommendation(s) using the following options (SELECT ALL THAT APPLY):**

□ Recommendation to change medication
□ Recommendation to decrease medication dose
□ Recommendation to increase medication dose
□ Recommendation to change medication timing of administration
□ Recommendation to start medication
□ Recommendation to discontinue medication
□ Recommendation to start monitoring

In the space below, SPECIFY the medication name/parameter that coincides with your recommendations noted above:

**Key:** NKDA = no known drug allergies; PO = by mouth; qAM = every morning; mg = milligram; qPM = every evening; ER = extended release; prn = as needed; TID = three times daily; BID = two times daily; q = every; h = hour.

## References

[1] Doan J., Zakrzewski-Jakubiak H., Roy J., Turgeon J., Tannenbaum C. (2013). Prevalence and Risk of Potential Cytochrome P450–Mediated Drug-Drug Interactions in Older Hospitalized Patients with Polypharmacy. Ann. Pharmacother..

[2] Mousavi S., Norouzi M., Ashouri A., Javadi M., Gholami K., Hadjibabaie M. (2014). Study of Potential Drug-Drug Interactions in Prescriptions of University-Based Pharmacies. J. Pharm. Care.

[3] Weideman R.A., Bernstein I.H., McKinney W.P. (1999). Pharmacist recognition of potential drug interactions. Am. J. Health Syst. Pharm..

[4] Agency for Healthcare Research and Quality. https://www.ahrq.gov/cpi/about/otherwebsites/clinical-decision-support/index.html.

[5] Jia P., Zhang L., Chen J., Zhao P., Zhang M. (2016). The Effects of Clinical Decision Support Systems on Medication Safety: An Overview. PLoS ONE.

[6] Turgeon J., Michaud V. (2016). Clinical Decision Support Systems: Great Promises for Better Management of Patients’ Drug Therapy. Expect Opin. Drug Metab. Toxicol..

[7] Zakrzewski-Jakubiak H., Doan J., Lamoureux P., Singh D., Turgeon J., Tannenbaum C. (2011). Detection and prevention of drug-drug interactions in the hospitalized elderly: Utility of new cytochrome p450-based software. Am. J. Geriatr. Pharmacother..

[8] US Trademark Registrations. https://uspto.report/TM/88768328.

[9] Survey Monkey. https://www.surveymonkey.com/.

[10] Systematized Nomenclature of Medicine—Clinical Terms. http://www.snomed.org/.

[11] Bankes D., Jin H., Finnel S., Michaud V., Knowlton C., Turgeon J., Stein A. (2020). Association of a Novel Medication Risk Score with Adverse Drug Events and Other Pertinent Outcomes Among Participants of the Programs of All-Inclusive Care for the Elderly. Pharmacy.

[12] Bain K., McGain D., Cicali E., Knowlton C., Michaud V., Turgeon J. (2019). Precision medication: An illustrative case series guiding the clinical application of multi-drug interactions and pharmacogenomics. Clin. Case Rep..

[13] Fowler S., Morcos P.N., Cleary Y., Martin-Facklam M., Parrott N., Gertz M., Yu L. (2017). Progress in Prediction and Interpretation of Clinically Relevant Metabolic Drug-Drug Interactions: A Minireview Illustrating Recent Developments and Current Opportunities. Curr. Pharmacol. Rep..

